# Global observational analysis of the Quality of Care Index (QCI) for protein-energy malnutrition from 1990 to 2021

**DOI:** 10.1097/MD.0000000000050041

**Published:** 2026-08-07

**Authors:** Yaxue Chen, Jia Liu, Min Deng, Wei Chen, Li Hu, Jingwen Zhang, Ying Ye, Hongjiang Pu

**Affiliations:** a Modern Nursing Department, Dazhou Vocational and Technical College, Dazhou, Sichuan, China; b Ultrasound Department, Dazhou Meinian Health Check-up Center, Dazhou, Sichuan, China; c School of Health Caring Industry, Sichuan University of Arts and Science, Dazhou, Sichuan, China; d Radiology Department, The Third Affiliated Hospital of Kunming Medical University, Peking University Cancer Hospital Yunnan Hospital, Yunnan Cancer Hospital, Kunming, China; e Department of Oncology, Dazhou Central Hospital, Dazhou, Sichuan, China.

**Keywords:** GBD, global burden, protein-energy malnutrition, QCI trends, quality of care index (QCI), SHAP analysis

## Abstract

Protein-energy malnutrition (PEM) remains a critical global health challenge, and the quality of care for PEM remains a key focus in the current public health field. Systematically evaluating these disparities is essential for guiding public health policy. This study adopted a revised Quality of Care Index (QCI) to evaluate global PEM care performance from 1990 to 2021. This study is based on data from the Global Burden of Disease (GBD) 1990 to 2021. The 4 secondary indicators were integrated through principal component analysis (PCA) to construct the QCI. The machine learning interpretability tool SHAP (SHapley Additive exPlanations) was utilized to deeply analyze the key factors affecting the QCI. The Bayesian age-period-cohort (BAPC) model was employed to predict the QCI trends from 2022 to 2033. From 1990 to 2021, while PEM incidence and prevalence counts increased, age-standardized rates of prevalence, incidence, mortality, and disability-adjusted life years (DALYs) declined. The QCI revealed substantial geographical disparities, strongly correlated with the Socio-Demographic Index (SDI). High-income regions, notably Northern Europe, achieved the highest QCI scores, while low-income regions, such as parts of West Africa and Southeast Asia, recorded the lowest. The QCI followed a U-shaped curve across the lifespan, being highest in children under 5, lowest in adolescents (10–14 years), and rising again in older adulthood. Sex-based differences were minimal. SHAP analysis confirmed SDI, age, and time as major QCI determinants. Projections suggest a continued, albeit slow, global improvement in the QCI. The QCI serves as a robust composite metric for benchmarking PEM care. Our findings highlight that the quality of PEM care is inextricably linked to socioeconomic development and underscore the need for targeted interventions aimed at vulnerable age groups and underserved regions to mitigate disparities and elevate global care standards.

## 1. Introduction

Protein-energy malnutrition (PEM) represents a spectrum of disorders arising from a sustained deficiency of macronutrients, classically categorized as marasmus, kwashiorkor, and mixed forms.^[1]^ The underlying pathophysiology involves a complex interplay of systemic metabolic adaptation and eventual decompensation in response to chronic absolute or relative deficits in energy and protein. While initial homeostatic mechanisms strive to maintain metabolic equilibrium, prolonged nutrient deprivation leads to a cascade of sequelae, including sarcopenia, hypoproteinemic edema, immune dysfunction, and multi-organ failure, establishing a detrimental vicious cycle. This compromised state significantly heightens susceptibility to infections and increases overall mortality.^[2-5]^ Furthermore, PEM is increasingly recognized as a significant comorbidity and exacerbating factor in various chronic conditions, including atrial fibrillation (AF).^[6]^ The etiology of PEM is multifactorial, primarily stemming from inadequate intake, impaired absorption, excessive nutrient losses, or elevated metabolic demands,^[7]^ posing a substantial threat to global health.

Contrary to common perception, PEM is not confined to developing nations; it is a prevalent yet frequently underdiagnosed condition among both hospitalized and community-dwelling populations in developed countries.^[8]^ The adequate provision of protein and energy is fundamental to health, and PEM remains a critical public health issue, disproportionately affecting the health and survival of young children in impoverished regions worldwide.

In the clinical management of PEM, the quality of nursing care is a pivotal determinant of treatment efficacy and patient prognosis. Nursing quality, defined as the provision of appropriate, effective healthcare services that align with patient needs and improve outcomes,^[9]^ has been the focus of studies investigating influencing factors and improvement strategies in PEM care.^[10–12]^ The nursing role is multifaceted. In pediatric severe acute malnutrition, key responsibilities include strict adherence to feeding protocols, meticulous monitoring of vital signs and fluid balance, vigilant observation for complications such as refeeding syndrome, and providing essential family education and psychosocial support.^[13]^ For geriatric populations, core nursing measures encompass systematic nutritional screening using validated tools like the Mini Nutritional Assessment (MNA), identifying and addressing barriers to adequate intake (e.g., dysphagia, cognitive impairment, depression), developing individualized dietary plans, assisting with feeding, ensuring oral care, and fostering interdisciplinary collaboration.^[14]^

To standardize care, authoritative guidelines have been established. The World Health Organization (WHO) outlines a structured “10-step” protocol for the management of severe acute malnutrition in children,^[15]^ while the European Society for Clinical Nutrition and Metabolism (ESPEN) provides comprehensive clinical guidelines for screening, assessment, diagnosis, and management of malnutrition (including PEM) in adults and the elderly.^[16]^

Despite these established protocols, a comprehensive, quantitative assessment of the global standard of nursing care for PEM is lacking. This study aims to address this gap by employing a novel Quality of Care Index (QCI) to provide a robust, multidimensional evaluation of PEM nursing quality from 1990 to 2021. The findings are intended to inform and guide future strategies for the prevention and clinical management of PEM globally.

## 2. Materials and methods

### 2.1. Data sources and collection

This global analysis was based on data from the Global Burden of Disease (GBD), accessible via the Global Health Data Exchange (https://vizhub.healthdata.org/gbd-results/) platform. The GBD study synthesizes a wide array of data sources, including vital registration systems, household surveys, published literature, and hospital records, to produce comprehensive and comparable estimates of disease burden for 204 countries and territories.^[17]^

For the period 1990 to 2021, we collected epidemiological data on Protein-Energy Malnutrition (PEM). The extracted metrics included incidence, mortality, prevalence, years of life lost (YLLs), years lived with disability (YLDs), and disability-adjusted life years (DALYs). All estimates were stratified by age and sex. The GBD modeling process, which employs Bayesian meta-regression tools and ensemble models, generates estimates with 95% uncertainty intervals (UIs) to reflect the precision of the data.^[18]^

Population data and structures were derived from World Health Organization (WHO) standard population compositions^[19]^ and the official United Nations World Population Prospects, which incorporate census data and demographic projections.^[20]^

The Socio-Demographic Index (SDI) is a composite measure of a region’s development status, ranging from 0 to 1, based on lag-distributed income per capita, average educational attainment for individuals aged 15 and older, and the total fertility rate under the age of 25.^[21]^ Countries were categorized into 5 SDI quintiles: high, high-middle, middle, low-middle, and low.^[22]^

### 2.2. Quality of Care Index (QCI)

This study established 4 secondary ratios (YLR, DPR, MIR, PIR) derived from 6 primary metrics to indirectly evaluate nursing quality parameters. All raw ratios negatively associate with care quality: lower values indicate more effective nutritional and clinical intervention. All ratios were reversed before PCA to guarantee a positive correlation between the composite PCA score and care quality.

PCA generates objective weights proportional to indicator factor loadings on Dim1, avoiding subjective weighting bias.^[9]^ Dim1 (51.8–56.4% variance) mainly reflects fatal care burden (MIR loading = 0.92, DPR loading = 0.90), while Dim2 captures nonfatal disability epidemiology (YLR loading = 0.87). The 2 orthogonal dimensions separate care quality signals from inherent disease traits. The first principal component was standardized to a 0 to 100 QCI via min–max normalization, with higher scores representing superior care.

The YLR indicator captures the extent of life loss attributable to premature mortality and the reduction in quality of life due to disability. A higher YLR value signifies that a disease primarily leads to early death, whereas a lower value implies a more substantial impact on quality of life than on mortality risk. The DPR metric assesses the relationship between disease burden and population health status. An elevated DPR indicates that although a disease is less common, it imposes serious health consequences; conversely, a lower DPR suggests wider prevalence but relatively moderate health loss. MIR reflects disease severity through the ratio of mortality to incidence and also serves as an inverse proxy for nursing quality – lower MIR values correspond to more effective nursing interventions. Finally, PIR represents the relationship between disease chronicity and the frequency of new cases. A higher PIR is indicative of chronic disease progression, while a lower PIR suggests an acute condition characterized by faster recovery or shorter duration.


$$\begin{array}{c} YLR=\frac{YLLs}{YLDs}, \end{array}$$

$$\begin{array}{c} DPR=\frac{DALYs}{Prevalence}, \end{array}$$

$$\begin{array}{c} MLR=\frac{Mortality}{Incidence}, \end{array}$$

$$\begin{array}{c} PIR=\frac{Prevalence}{Incidence}. \end{array}$$

PCA a multivariate statistical technique, was employed to distill orthogonal components from the dataset. In this study, PCA was applied to integrate the 4 indicators into a reduced set of principal components. These components were then transformed into a standardized score ranging from 0 to 100, where higher values denote superior nursing quality, to facilitate a more intuitive comparison of differences.^[23]^ The specific calculation formula is as follows:


$$QCI\left(x\right)=\left(\frac{PCAscore\left(x\right)-minPCAscore\left(x\right)}{maxPCAscore\left(x\right)-minPCAscore\left(x\right)}\right)\times 100\%.$$

Notably, this QCI is constructed purely from population-level disease outcome metrics (YLLs, YLDs, mortality, incidence, and prevalence) due to data constraints. Globally standardized, long-term panel data on structural and process care indicators (including medical infrastructure, nutrition service accessibility, clinical staffing, and guideline adherence rates) are unavailable for all 204 countries and territories from 1990 to 2021, which restricts the integration of direct care process/structural variables into the composite index. This outcome-based construction follows established QCI frameworks for global GBD comparative research.^[9,23]^

### 2.3. SHAP analysis

This study first constructed an XGBoost regression model to predict QCI values, then adopted SHAP (SHapley Additive exPlanations) analysis to interpret model predictions. The dataset was randomly split into an 80% training set and a 20% independent test set. Five-fold cross-validation with grid search was performed for hyperparameter tuning, with finalized settings: learning rate = 0.01, max depth = 6, subsample = 0.8, n_estimators = 500. Model generalization was assessed via RMSE and R^2^ to avoid overfitting. After validation, TreeSHAP, a dedicated algorithm for tree-based models, was applied to compute SHAP values. Rooted in the Shapley value theory of cooperative games, SHAP quantifies each feature’s marginal contribution to individual predictions by averaging feature effects across all variable combinations. This framework delivers unified feature importance metrics, supporting both global interpretability (screening dominant predictors of QCI) and local interpretability (explaining single-sample prediction outputs), which offers transparent evidence to support our analytical conclusions.

### 2.4. Bayesian Age-Period-Cohort (BAPC) model for forecasting

To project future trends of the Quality of Care Index (QCI), we employed the Bayesian Age-Period-Cohort (BAPC) model. This approach simultaneously estimates the distinct effects of age, time period, and birth cohort while addressing the inherent identifiability issue through constrained parameterization.

Temporal smoothing for each component was achieved by assigning second-order random walk (RW2) priors. Parameter estimation and uncertainty quantification were conducted within a Bayesian inference framework, which provides a complete probabilistic characterization of future trajectories.

For the forecasting phase beyond the observed data (2022–2030), the model components were extrapolated as follows: period effects were projected using autoregressive integrated moving average (ARIMA) techniques, cohort effects were modeled based on established demographic transition patterns, and age effects were assumed to remain constant at their most recently estimated levels. This constraint resolves the collinearity issue intrinsic to BAPC models per standard GBD analytical protocols. Our forecast spans merely 12 years, during which core age-related clinical and physiological features of PEM are unlikely to undergo drastic changes; without robust covariates to support credible dynamic extrapolation of age trends, this approach avoids introducing arbitrary bias into projections. While age effects might shift over far longer horizons, we provided full 95% prediction intervals for all forecast results to reflect the uncertainty associated with this simplification.

Model computation was performed using integrated nested Laplace approximation (INLA) algorithms for efficient Bayesian inference. Model performance and selection were assessed through the deviance information criterion (DIC) and cross-validation procedures. This comprehensive methodological framework ensures robust long-term forecasting of QCI trends with fully quantified uncertainty.

All forecast outputs are accompanied by 95% prediction intervals derived from Bayesian posterior distributions, whose wide ranges reflect cumulative uncertainty from raw GBD data noise, model identifiability constraints, and long-term trend extrapolation error.

## 3. Statistical analysis

All statistical analyses and data visualizations were performed using R (version 4.4.2; R Foundation for Statistical Computing). Descriptive statistics were generated for all key variables, and results were expressed as means with 95% uncertainty intervals (UIs). For trend analyses, *P*-values < .05 were considered statistically significant.

## 4. Ethical statement

The data used in this study were all obtained from public sources and did not involve any confidential or personal information.

## 5. Result

### 5.1. Global burden of protein-energy malnutrition

From 1990 to 2021, the total number of incident cases of global protein-energy malnutrition (PEM) increased from 93.72 million (95% UI: 75.37–117.21 million) to 108.86 million cases (91.32–130.57 million; Table 1). The number of prevalence cases increased from 113.02 million (104.58–123.62 million) in 1990 to 127.22 million cases (115.16–142.91 million) in 2021 (Table 2). The number of deaths decreased significantly, from 5,05,632 cases (4,29,698–6,19,495) in 1990 to 1,89,346 cases (1,67,551–2,13,755) in 2021 (Table 3). DALYs fell from 42.0 million (95% UI: 35.5–52.5 million) to 11.8 million (95% UI: 9.9–14.0 million; Table 4). The age-standardized prevalence, incidence, mortality, and disability-adjusted life year (DALY) rates of protein-energy malnutrition (PEM) worldwide showed a downward trend.

**Table 1:** Number of cases and incidence rates of protein-energy malnutrition, in 5 SDI regions and 21 geographical regions from 1990 to 2021.

Protein-energy malnutrition					
Location	Incident cases		Incident rate		EAPC
	1990	2021	1990	2021	1990–2021
Global	9,37,17,000.65 (7,53,65,559.18, 11,72,09,681.50)	10,88,57,028.31 (9,13,19,665.29, 13,05,70,454.18)	1677.02 (1363.96, 2070.23)	1406.21 (1177.88, 1691.07)	0.28 (−0.44, −0.12)
High SDI	93,64,422.96 (74,85,915.57, 1,16,03,761.67)	1,42,41,079.88 (1,19,04,536.86, 1,69,25,229.80)	1052.68 (828.38, 1301.59)	1118.15 (931.89, 1329.48)	0.61 (0.31, 0.92)
High-middle SDI	1,04,96,655.48 (82,15,977.31, 1,30,95,568.02)	1,69,11,199.93 (1,41,21,431.04, 2,03,79,222.85)	1026.36 (808.06, 1286.82)	1202.77 (996.16, 1464.96)	0.80 (0.54, 1.06)
Middle SDI	2,77,08,736.98 (2,22,99,196.83, 3,45,27,080.25)	3,49,44,753.22 (2,92,56,722.47, 4,24,76,578.25)	1584.92 (1292.55, 1965.10)	1445.72 (1205.61, 1752.87)	−0.01 (−0.24, 0.23)
Low-middle SDI	3,15,08,831.87 (2,45,47,039.96, 3,93,19,911.86)	2,70,30,048.49 (2,22,60,599.25, 3,24,83,449.19)	2260.85 (1824.50, 2778.40)	1447.85 (1198.87, 1736.67)	−1.22 (−1.41, −1.03)
Low SDI	1,45,83,127.32 (1,13,29,098.09, 1,83,35,918.60)	1,56,64,550.08 (1,24,87,198.10, 1,95,06,526.28)	2134.82 (1752.88, 2595.12)	1260.03 (1043.98, 1516.13)	−1.34 (−1.53, −1.14)
Andean Latin America	2,59,162.82 (2,24,555.40, 2,93,770.20)	3,90,092.15 (3,11,545.52, 4,59,591.83)	729.91 (643.85, 818.05)	603.80 (482.44, 710.52)	−0.71 (−0.81, −0.61)
Australasia	1,44,937.99 (1,16,616.82, 1,81,168.63)	2,72,509.34 (2,36,525.31, 3,26,133.84)	701.10 (564.36, 880.62)	755.20 (650.47, 909.65)	0.45 (0.21, 0.69)
Caribbean	3,05,161.50 (2,59,318.64, 3,55,393.42)	2,86,393.75 (2,48,917.47, 3,28,217.83)	861.17 (740.04, 995.34)	611.98 (528.47, 706.43)	−0.81 (−0.93, −0.69)
Central Asia	7,02,261.78 (5,23,132.58, 9,16,462.73)	7,16,914.90 (5,64,424.26, 8,93,761.66)	859.35 (650.88, 1106.48)	741.53 (585.62, 924.03)	−1.02 (−1.17, −0.86)
Central Europe	7,96,655.57 (5,99,643.99, 10,14,994.53)	10,59,940.00 (8,69,438.96, 13,00,193.60)	697.64 (529.02, 886.70)	893.05 (714.88, 1111.43)	1.03 (0.78, 1.27)
Central Latin America	19,31,602.03 (16,32,899.97, 23,02,021.68)	21,46,011.00 (18,97,647.99, 24,56,976.54)	1257.66 (1085.38, 1475.86)	846.10 (746.17, 967.91)	−0.58 (−0.91, −0.25)
Central Sub-Saharan Africa	10,62,459.11 (8,04,983.41, 13,46,600.88)	10,73,984.95 (8,30,611.82, 13,34,751.44)	1379.40 (1129.17, 1649.22)	733.42 (610.86, 870.69)	−1.79 (−2.13, −1.45)
East Asia	1,45,01,969.80 (1,15,61,506.26, 1,82,01,537.95)	2,26,35,288.13 (1,85,42,821.46, 2,74,33,506.00)	1243.43 (992.17, 1558.93)	1379.46 (1129.10, 1698.95)	0.55 (0.23, 0.87)
Eastern Europe	18,87,482.52 (14,55,493.28, 23,97,474.59)	14,53,522.70 (11,25,596.23, 18,26,749.87)	934.12 (713.25, 1182.54)	815.12 (623.72, 1024.76)	−0.36 (−0.58, −0.13)
Eastern Sub-Saharan Africa	44,73,815.18 (36,11,697.42, 54,77,526.61)	42,10,141.12 (34,16,182.76, 50,48,989.19)	1866.44 (1589.94, 2223.22)	933.22 (795.36, 1076.96)	−1.92 (−2.04, −1.80)
High-income Asia Pacific	18,41,702.74 (14,13,305.81, 23,57,867.35)	19,69,611.76 (16,40,063.21, 23,78,648.80)	1122.07 (868.59, 1431.74)	966.95 (796.81, 1197.99)	−0.29 (−0.51, −0.07)
High-income North America	33,12,843.61 (25,58,905.90, 43,04,280.07)	47,87,259.51 (38,27,306.53, 59,15,508.12)	1114.90 (857.90, 1440.76)	1083.83 (853.64, 1340.40)	0.27 (−0.09, 0.62)
North Africa and Middle East	51,26,771.49 (42,09,327.17, 62,51,410.31)	65,24,401.76 (53,74,388.54, 78,66,330.47)	1288.36 (1069.03, 1570.28)	1074.50 (891.92, 1290.55)	−0.48 (−0.75, −0.22)
Oceania	1,26,516.91 (1,02,097.65, 1,53,498.14)	2,36,436.16 (1,91,004.49, 2,82,547.32)	1789.31 (1489.60, 2115.97)	1515.60 (1278.41, 1752.19)	−0.14 (−0.43, 0.15)
South Asia	3,62,73,730.54 (2,76,76,210.79, 4,59,82,456.56)	3,57,74,684.33 (2,89,28,325.62, 4,39,13,325.28)	2762.86 (2178.39, 3458.38)	2044.84 (1655.49, 2494.51)	−0.66 (−0.86, −0.46)
Southeast Asia	1,03,19,793.75 (82,80,080.71, 1,27,56,671.29)	1,01,84,365.55 (87,78,262.45, 1,18,59,308.77)	2128.86 (1764.35, 2623.18)	1502.74 (1293.10, 1744.65)	−0.87 (−0.97, −0.76)
Southern Latin America	3,10,458.05 (2,66,283.68, 3,60,366.84)	4,94,862.00 (4,19,518.31, 5,82,050.07)	635.47 (546.42, 737.58)	675.20 (574.01, 797.06)	−0.35 (−0.60, −0.11)
Southern Sub-Saharan Africa	5,59,007.43 (4,33,271.68, 7,16,679.34)	5,00,078.75 (4,30,863.01, 5,79,530.60)	943.91 (758.42, 1187.77)	650.59 (563.12, 749.90)	−1.08 (−1.35, −0.81)
Tropical Latin America	16,56,125.08 (13,48,841.65, 20,92,785.07)	21,89,483.21 (18,62,278.85, 25,77,497.91)	1198.31 (986.35, 1486.77)	905.53 (768.46, 1066.43)	0.25 (−0.20, 0.69)
Western Europe	35,39,870.93 (28,54,198.68, 43,60,053.04)	62,89,443.05 (52,34,410.15, 75,21,133.31)	890.36 (706.16, 1118.06)	1160.03 (958.17, 1404.49)	1.44 (1.12, 1.77)
Western Sub-Saharan Africa	45,84,671.82 (35,45,321.94, 58,23,340.87)	56,61,604.18 (42,35,669.47, 72,08,759.63)	1596.47 (1294.28, 1950.39)	918.79 (740.40, 1126.77)	−1.23 (−1.48, −0.98)

EAPC is expressed as a 95% confidence interval.

EAPC = estimated annual percentage change, SDI = Socio-Demographic Index, UI = uncertainty interval.

**Table 2:** Number of cases and prevalence rates of protein-energy malnutrition, in 5 SDI regions and 21 geographical regions from 1990 to 2021.

Protein-energy malnutrition					
Location	Prevalence cases		Prevalence rate		EAPC
	1990	2021	1990	2021	1990–2021
Global	11,30,16,684.38 (10,45,75,661.51, 12,36,15,655.34)	12,72,18,221.69 (11,51,60,546.28, 14,29,11,202.71)	1986.52 (1827.52, 2186.62)	1696.86 (1548.51, 1892.08)	−0.28 (−0.44, −0.12)
High SDI	91,36,788.07 (76,75,945.99, 1,10,02,625.89)	1,41,04,439.30 (1,20,00,934.65, 1,65,64,544.97)	1038.30 (873.79, 1255.65)	1090.94 (934.79, 1284.68)	0.61 (0.31, 0.92)
High-middle SDI	1,14,57,537.11 (1,00,08,114.44, 1,33,18,961.20)	1,68,83,117.54 (1,45,61,249.61, 1,98,68,832.40)	1139.83 (1005.93, 1310.89)	1227.48 (1071.70, 1428.90)	0.80 (0.54, 1.06)
Middle SDI	3,29,81,444.99 (3,01,94,679.56, 3,64,01,732.79)	3,80,08,748.79 (3,37,17,547.33, 4,36,98,924.45)	1847.30 (1685.19, 2052.26)	1647.25 (1480.24, 1871.05)	−0.01 (−0.24, 0.23)
Low-middle SDI	4,04,79,239.32 (3,82,43,294.76, 43,16,8613.10)	3,49,48,219.26 (3,22,33,453.50, 3,84,74,038.86)	2763.59 (2566.34, 3003.69)	1871.94 (1723.90, 2054.95)	−1.22 (−1.41, −1.03)
Low SDI	1,88,97,839.17 (1,81,93,949.06, 1,97,57,082.19)	2,31,99,383.10 (2,21,67,001.57, 2,44,47,188.46)	2599.43 (2449.53, 2788.37)	1708.11 (1611.49, 1832.18)	−1.34 (−1.53, −1.14)
Andean Latin America	2,84,632.53 (2,65,732.26, 3,05,491.26)	3,96,582.15 (3,27,294.13, 4,57,141.69)	772.18 (712.31, 839.46)	621.20 (514.26, 715.34)	−0.71 (−0.81, −0.61)
Australasia	1,38,744.08 (1,17,369.16, 1,67,743.44)	2,69,860.64 (2,35,308.97, 3,18,672.80)	671.53 (569.93, 812.05)	734.17 (636.16, 872.99)	0.45 (0.21, 0.69)
Caribbean	3,54,815.10 (3,28,441.45, 3,83,967.68)	3,44,742.96 (3,21,822.04, 3,76,622.06)	983.04 (906.52, 1072.29)	766.48 (720.05, 831.10)	−0.81 (−0.93, −0.69)
Central Asia	9,10,266.04 (8,35,752.25, 9,90,807.91)	7,81,377.61 (6,85,358.25, 9,01,290.46)	1072.65 (974.90, 1191.04)	806.46 (707.16, 930.90)	−1.02 (−1.17, −0.86)
Central Europe	8,63,533.09 (7,26,576.32, 10,37,013.39)	10,67,318.44 (9,15,632.86, 12,58,653.93)	781.73 (671.34, 922.02)	925.13 (793.69, 1090.97)	1.03 (0.78, 1.27)
Central Latin America	20,85,941.96 (19,02,517.44, 23,26,803.45)	22,04,998.05 (19,88,567.20, 24,90,153.52)	1313.89 (1186.21, 1487.17)	890.29 (806.73, 999.73)	−0.58 (−0.91, −0.25)
Central Sub-Saharan Africa	15,35,289.45 (14,79,373.47, 15,93,492.15)	18,75,299.35 (17,87,612.39, 19,64,684.17)	1835.94 (1737.37, 1942.07)	1108.72 (1044.31, 1178.45)	−1.79 (−2.13, −1.45)
East Asia	1,56,16,053.86 (1,36,86,013.24, 1,80,88,392.94)	2,21,46,428.92 (1,86,56,480.27, 2,64,41,536.67)	1352.16 (1192.58, 1561.97)	1349.27 (1149.08, 1614.85)	0.55 (0.23, 0.87)
Eastern Europe	21,56,242.95 (18,38,853.02, 25,26,919.64)	14,75,868.47 (12,13,480.25, 17,69,527.40)	1102.21 (959.59, 1271.75)	873.50 (741.31, 1014.02)	−0.36 (−0.58, −0.13)
Eastern Sub-Saharan Africa	53,45,446.41 (51,09,758.17, 56,27,530.38)	59,36,602.08 (57,45,611.66, 61,70,831.06)	2086.62 (1942.84, 2267.07)	1198.73 (1144.88, 1269.47)	−1.92 (−2.04, −1.80)
High-income Asia Pacific	17,42,157.64 (14,19,449.75, 21,58,450.98)	19,63,755.51 (16,64,374.17, 23,32,582.68)	1066.58 (886.62, 1303.11)	945.80 (811.72, 1118.15)	−0.29 (−0.51, −0.07)
High-income North America	31,41,739.88 (25,05,527.76, 39,70,357.02)	47,02,225.30 (37,70,336.87, 57,38,837.48)	1053.55 (835.92, 1334.29)	1038.32 (823.32, 1262.57)	0.27 (−0.09, 0.62)
North Africa and Middle East	68,22,193.55 (63,83,055.34, 74,08,145.59)	74,63,576.57 (67,55,526.58, 82,94,871.75)	1606.46 (1479.24, 1781.03)	1234.68 (1123.34, 1365.96)	−0.48 (−0.75, −0.22)
Oceania	1,47,888.92 (1,36,882.75, 1,60,046.23)	2,80,281.85 (2,63,332.72, 2,97,621.48)	1998.71 (1809.80, 2202.77)	1752.16 (1635.62, 1875.08)	−0.14 (−0.43, 0.15)
South Asia	4,74,02,685.27 (4,48,09,392.11, 5,05,34,454.14)	4,58,68,531.76 (4,15,11,134.50, 5,15,28,411.69)	3452.67 (3211.15, 3746.86)	2712.28 (2484.04, 3008.72)	−0.66 (−0.86, −0.46)
Southeast Asia	1,23,97,052.61 (1,15,55,431.64, 1,33,97,276.88)	1,19,45,382.78 (1,09,57,283.97, 1,32,04,402.31)	2482.51 (2294.00, 2736.32)	1841.07 (1704.55, 2014.54)	−0.87 (−0.97, −0.76)
Southern Latin America	3,58,630.15 (3,27,925.55, 3,98,189.44)	4,91,077.06 (4,18,124.14, 5,71,901.39)	729.88 (666.73, 809.18)	677.81 (585.84, 779.30)	−0.35 (−0.60, −0.11)
Southern Sub-Saharan Africa	6,54,047.38 (6,10,560.71, 7,07,454.34)	5,83,684.03 (5,39,225.92, 6,36,628.71)	1065.29 (979.32, 1174.31)	759.24 (700.93, 827.64)	−1.08 (−1.35, −0.81)
Tropical Latin America	18,10,329.03 (15,59,944.02, 21,33,352.07)	22,20,284.12 (19,24,229.04, 26,00,209.82)	1294.07 (1117.43, 1525.52)	936.76 (818.54, 1087.48)	0.25 (−0.20, 0.69)
Western Europe	34,18,946.10 (28,95,058.87, 41,00,002.41)	62,83,477.29 (52,81,387.80, 74,02,314.40)	863.94 (723.72, 1042.39)	1130.92 (958.72, 1342.85)	1.44 (1.12, 1.77)
Western Sub-Saharan Africa	58,30,048.37 (56,31,993.49, 60,60,600.77)	89,16,866.73 (86,09,211.41, 92,44,967.67)	1959.30 (1850.32, 2096.04)	1324.58 (1265.81, 1392.56)	−1.23 (−1.48, −0.98)

EAPC is expressed as a 95% confidence interval.

EAPC = estimated annual percentage change, SDI = sociodemographic Index, UI = uncertainty interval.

**Table 3:** Number of cases and mortality rates of protein-energy malnutrition in 5 SDI regions and 21 geographical regions from 1990 to 2021.

Protein-energy malnutrition					
	Deaths cases		Mortality rate		EAPC
Location	1990	2021	1990	2021	1990–2021
Global	5,05,632.39 (4,29,698.14, 6,19,494.57)	1,89,345.50 (1,67,550.85, 2,13,754.71)	9.44 (8.16, 11.32)	2.61 (2.29, 2.98)	−4.47 (−4.80, −4.13)
High SDI	6279.00 (5645.73, 6689.91)	22,690.73 (18,740.84, 24,816.61)	0.63 (0.57, 0.68)	0.88 (0.74, 0.95)	0.58 (0.03, 1.13)
High-middle SDI	14,947.49 (13,498.96, 16,495.06)	10,613.14 (9185.86, 11,820.55)	1.81 (1.63, 1.99)	0.63 (0.55, 0.69)	−3.70 (−4.07, −3.33)
Middle SDI	95,700.06 (88,274.63, 1,04,633.81)	43,773.02 (39,495.80, 47,443.51)	8.45 (7.76, 9.03)	2.17 (1.94, 2.35)	−4.38 (−4.46, −4.29)
Low-middle SDI	1,93,310.29 (1,60,716.65, 2,37,297.89)	39,413.38 (34,086.99, 44,856.85)	14.96 (12.54, 17.93)	2.74 (2.40, 3.07)	−6.38 (−7.24, −5.52)
Low SDI	1,95,101.12 (1,51,596.64, 2,57,103.40)	72,689.97 (56,079.00, 88,933.24)	31.07 (25.80, 38.10)	7.68 (6.44, 8.91)	−4.38 (−5.08, −3.67)
Andean Latin America	6507.58 (5645.37, 7518.22)	2665.93 (2216.14, 3252.27)	20.31 (18.11, 22.68)	4.69 (3.90, 5.72)	−5.11 (−5.39, −4.82)
Australasia	50.93 (46.59, 54.43)	137.26 (114.77, 151.73)	0.24 (0.21, 0.25)	0.22 (0.19, 0.24)	−0.57 (−0.95, −0.19)
Caribbean	3819.92 (3198.46, 4562.84)	1761.54 (1414.39, 2240.85)	10.51 (8.99, 12.35)	3.90 (3.08, 5.07)	−3.04 (−3.43, −2.65)
Central Asia	505.05 (453.01, 555.52)	152.30 (132.90, 174.10)	0.66 (0.60, 0.72)	0.18 (0.16, 0.21)	−5.21 (−5.74, −4.68)
Central Europe	130.97 (120.91, 143.66)	661.55 (603.44, 715.57)	0.11 (0.10, 0.13)	0.32 (0.29, 0.35)	2.78 (1.99, 3.57)
Central Latin America	22,833.81 (21,862.81, 23,847.62)	10,568.25 (9472.21, 11,756.09)	22.56 (21.57, 23.30)	4.60 (4.12, 5.12)	−5.24 (−5.34, −5.14)
Central Sub-Saharan Africa	23,592.47 (17,431.60, 34,371.75)	8832.39 (6090.02, 12,081.94)	33.56 (26.94, 45.34)	9.33 (6.89, 12.22)	−4.34 (−4.71, −3.97)
East Asia	36,036.81 (31,272.40, 41,382.45)	13,106.98 (10,880.21, 15,308.71)	5.15 (4.54, 5.75)	0.91 (0.75, 1.06)	−8.85 (−11.16, −6.48)
Eastern Europe	509.61 (480.24, 537.81)	421.41 (394.02, 449.32)	0.23 (0.21, 0.24)	0.15 (0.14, 0.15)	−2.69 (−3.83, −1.54)
Eastern Sub-Saharan Africa	1,16,482.37 (91,731.07, 1,52,262.19)	41,314.71 (32,961.71, 50,118.50)	54.91 (46.55, 65.45)	13.42 (11.54, 15.55)	−4.44 (−5.40, −3.48)
High-income Asia Pacific	583.24 (523.98, 626.28)	1811.87 (1501.48, 1990.15)	0.34 (0.30, 0.37)	0.33 (0.29, 0.35)	−0.43 (−0.79, −0.06)
High-income North America	2048.36 (1802.06, 2173.20)	13,039.78 (10,915.35, 14,216.59)	0.56 (0.50, 0.60)	1.77 (1.50, 1.91)	2.90 (1.97, 3.84)
North Africa and Middle East	13,034.43 (10,105.41, 18,871.26)	4342.68 (3639.22, 5157.97)	3.52 (2.89, 4.69)	1.01 (0.86, 1.18)	−4.19 (−4.34, −4.04)
Oceania	237.51 (189.65, 296.40)	292.25 (226.49, 377.77)	7.56 (6.28, 9.08)	4.35 (3.56, 5.40)	−1.83 (−1.88, −1.79)
South Asia	1,68,755.52 (1,35,019.17, 2,08,100.51)	16,803.86 (13,186.74, 21,283.68)	12.73 (10.14, 15.77)	1.15 (0.90, 1.44)	−7.29 (−7.53, −7.05)
Southeast Asia	36,889.38 (30,745.50, 44,460.72)	25,467.99 (21,737.35, 28,700.68)	13.23 (11.16, 15.08)	5.22 (4.44, 5.87)	−2.80 (−2.96, −2.63)
Southern Latin America	1862.62 (1771.60, 1952.92)	1357.21 (1197.21, 1472.71)	4.09 (3.89, 4.30)	1.55 (1.38, 1.68)	−3.11 (−3.78, −2.43)
Southern Sub-Saharan Africa	7319.79 (6144.37, 8851.18)	5637.46 (4543.57, 6888.70)	12.59 (10.95, 14.64)	8.09 (6.62, 9.74)	−0.73 (−0.99, −0.47)
Tropical Latin America	12,058.04 (11,170.62, 13,021.10)	5774.70 (5051.13, 6228.23)	10.37 (9.65, 11.03)	2.44 (2.14, 2.64)	−4.74 (−5.04, −4.44)
Western Europe	2815.72 (2476.68, 3050.87)	7661.62 (6149.06, 8639.84)	0.53 (0.46, 0.57)	0.58 (0.47, 0.65)	0.34 (0.14, 0.54)
Western Sub-Saharan Africa	49,558.27 (37,217.92, 68,229.57)	27,533.77 (19,572.99, 35,578.31)	18.52 (14.86, 23.69)	6.22 (4.97, 7.53)	−3.44 (−3.56, −3.31)

EAPC is expressed as a 95% confidence interval.

EAPC = estimated annual percentage change, SDI = Socio-Demographic Index, UI = uncertainty interval.

**Table 4:** Number of cases and DALY rates of protein-energy malnutrition in 5 SDI regions and 21 geographical regions from 1990 to 2021.

Protein-energy malnutrition					
Location	DALYs cases		DALYs rate		EAPC
	1990	2021	1990	2021	1990–2021
Global	4,20,12,720.56 (3,55,49,209.61, 5,25,08,991.25)	1,18,26,130.80 (98,81,913.81, 1,39,63,539.45)	699.30 (594.81, 869.67)	172.38 (142.75, 204.10)	−4.64 (−4.97, −4.30)
High SDI	1,84,404.73 (1,50,081.29, 2,45,271.76)	5,73,108.16 (4,50,879.07, 7,67,109.15)	21.66 (17.72, 28.31)	34.33 (25.63, 48.18)	1.86 (1.70, 2.03)
High-middle SDI	1,007,923.75 (8,99,847.46, 11,36,164.32)	2,79,114.68 (2,40,898.89, 3,34,866.89)	110.29 (98.33, 124.30)	22.49 (19.18, 26.71)	−5.46 (−5.75, −5.16)
Middle SDI	70,36,064.12 (63,92,350.52, 79,03,869.11)	18,48,150.90 (16,11,310.49, 21,31,866.90)	402.70 (368.31, 446.62)	91.34 (79.47, 105.43)	−4.62 (−4.71, −4.52)
Low-middle SDI	1,71,53,040.74 (14,409,706.38, 2,08,35,603.26)	31,63,670.89 (26,80,398.69, 37,25,985.78)	1058.61 (892.98, 1279.44)	175.06 (148.99, 204.94)	−6.25 (−6.87, −5.63)
Low SDI	1,66,08,624.72 (1,28,97,552.09, 2,21,27,284.71)	59,53,067.29 (45,26,049.00, 73,39,801.19)	2019.51 (1602.83, 2637.16)	424.78 (333.52, 512.50)	−4.79 (−5.47, −4.10)
Andean Latin America	4,28,980.80 (3,59,463.97, 5,12,886.96)	85,315.36 (67,041.83, 1,06,301.28)	931.39 (795.28, 1091.49)	141.25 (111.60, 176.05)	−6.41 (−6.73, −6.08)
Australasia	981.08 (915.42, 1034.23)	2047.52 (1660.30, 3416.14)	4.55 (4.25, 4.81)	4.01 (3.22, 7.77)	−0.67 (−1.07, −0.27)
Caribbean	2,99,127.84 (2,45,334.39, 3,63,339.55)	99,789.56 (74,754.06, 1,36,157.26)	735.17 (606.98, 888.45)	246.64 (182.65, 339.20)	−3.29 (−3.64, −2.93)
Central Asia	58,361.59 (49,738.24, 68,234.64)	12,226.42 (10,126.23, 14,695.38)	66.73 (57.40, 77.58)	12.71 (10.59, 15.29)	−6.35 (−6.85, −5.84)
Central Europe	10,711.37 (8935.80, 12,918.83)	14,542.08 (13,352.08, 15,694.71)	10.75 (8.87, 13.07)	9.70 (8.80, 10.65)	−1.05 (−1.58, −0.52)
Central Latin America	12,30,234.91 (11,58,104.09, 13,18,011.99)	3,08,053.03 (2,67,398.36, 3,60,190.29)	728.51 (695.69, 766.47)	136.93 (117.23, 162.22)	−5.36 (−5.46, −5.27)
Central Sub-Saharan Africa	19,88,448.83 (14,51,630.65, 29,18,072.66)	6,45,548.21 (4,23,914.92, 9,02,526.71)	2117.78 (1600.17, 3022.55)	409.82 (292.45, 548.20)	−5.36 (−5.83, −4.88)
East Asia	25,54,266.32 (21,57,794.13, 30,02,060.79)	2,25,144.98 (1,91,243.85, 2,65,079.10)	241.67 (207.45, 279.94)	17.67 (15.15, 20.36)	−12.53 (−15.28, −9.70)
Eastern Europe	62,179.79 (48,331.20, 78,109.20)	25,247.95 (20,549.66, 31,074.70)	32.25 (24.89, 40.89)	14.10 (10.92, 18.11)	−2.91 (−3.31, −2.51)
Eastern Sub-Saharan Africa	94,05,538.80 (73,18,613.47, 1,25,20,422.10)	30,08,475.31 (23,13,932.01, 37,48,599.42)	3063.40 (2456.21, 3955.91)	602.35 (487.42, 724.98)	−5.03 (−6.02, −4.03)
High-income Asia Pacific	16,598.06 (15,247.00, 18,123.54)	29,856.97 (26,541.56, 31,893.58)	9.72 (8.88, 10.72)	8.49 (7.90, 9.13)	−0.73 (−1.06, −0.39)
High-income North America	59,834.50 (34,593.52, 1,15,681.04)	3,58,146.77 (2,79,960.12, 4,68,890.13)	18.50 (10.08, 37.11)	66.79 (49.56, 90.93)	3.90 (3.40, 4.40)
North Africa and Middle East	12,57,956.88 (9,99,621.86, 17,75,417.51)	3,55,790.15 (2,87,439.49, 4,37,678.09)	268.66 (216.64, 369.83)	62.12 (50.66, 76.10)	−4.75 (−5.03, −4.47)
Oceania	18,618.26 (14,781.62, 22,795.50)	23,565.33 (17,936.21, 30,151.56)	268.03 (222.04, 322.61)	167.02 (131.17, 209.58)	−1.34 (−1.53, −1.16)
South Asia	1,62,74,236.86 (1,33,70,995.81, 1,97,02,978.29)	26,70,739.98 (20,75,812.62, 33,75,235.19)	1083.59 (891.43, 1310.04)	165.27 (128.86, 207.35)	−5.61 (−5.81, −5.40)
Southeast Asia	24,76,029.26 (20,48,253.47, 31,69,530.90)	8,84,092.36 (7,73,332.74, 10,08,287.61)	534.62 (451.28, 654.60)	153.01 (134.56, 173.40)	−3.90 (−4.01, −3.78)
Southern Latin America	99,145.41 (93,659.16, 1,04,823.91)	28,822.24 (23,113.71, 37,311.99)	200.18 (189.14, 211.43)	39.08 (31.65, 49.77)	−5.20 (−5.68, −4.73)
Southern Sub-Saharan Africa	5,90,343.24 (4,87,059.73, 7,28,368.31)	3,94,898.92 (3,07,298.90, 4,90,546.57)	856.31 (715.65, 1037.42)	510.16 (399.75, 631.88)	−0.68 (−1.08, −0.27)
Tropical Latin America	8,25,255.70 (7,45,676.27, 9,10,684.39)	1,47,950.45 (1,35,456.93, 1,59,723.97)	552.82 (504.24, 605.50)	66.47 (60.22, 72.77)	−7.09 (−7.46, −6.72)
Western Europe	41,833.64 (36,647.10, 51,749.14)	1,74,554.73 (1,03,735.38, 2,90,109.43)	8.61 (7.53, 10.83)	22.63 (10.89, 41.13)	4.20 (3.82, 4.58)
Western Sub-Saharan Africa	43,14,037.42 (32,46,048.48, 59,66,617.95)	23,31,322.48 (16,61,253.20, 30,25,492.11)	1285.88 (982.53, 1750.29)	339.84 (250.12, 431.22)	−4.15 (−4.32, −3.98)

EAPC is expressed as a 95% confidence interval.

DALY = disability-adjusted life years, EAPC = estimated annual percentage change, SDI = Socio-Demographic Index, UI = uncertainty interval.

There are significant differences in the disease burden across different Socio-Demographic Index (SDI) regions. A clear inverse relationship was observed between development level and the magnitude of absolute case reduction. The most substantial improvements occurred in low and low-middle SDI regions. Deaths in low SDI regions fell from 1,95,101 (UI: 1,51,596–2,57,103) to 72,689 (UI: 56,079–88,933), while low-middle SDI regions saw a dramatic decline from 1,93,310 (UI: 1,60,717–2,37,298) to 39,413 (UI: 34,087–44,857). Middle and high-middle SDI regions also recorded significant mortality reductions (Table 3).

Conversely, a positive correlation was found between development level and the growth in case counts. High and high-middle SDI regions experienced marked increases in both incident and prevalent cases. The number of incident cases in high SDI regions rose from 9.36 million to 14.24 million, and prevalent cases grew from 9.14 million to 14.1 million. A similar upward trend was observed in high-middle SDI regions. Meanwhile, middle SDI regions saw a moderate increase in cases, and low-middle SDI regions experienced a decrease (Tables 1 and 2).

The high SDI region was the only group to experience an increase in both DALY count and rate. In contrast, all other SDI regions exhibited notable declines (Table 4).

This divergent trend – increasing case counts in high-SDI regions alongside rising mortality in these same areas, against a backdrop of sharply declining mortality in low-SDI regions – highlights a shifting global burden of PEM and underscores the critical influence of socioeconomic determinants on the disease’s epidemiological profile.

### 5.2. QCI result

#### 5.2.1. QCI trends in various countries around the world

**Figure 1.**
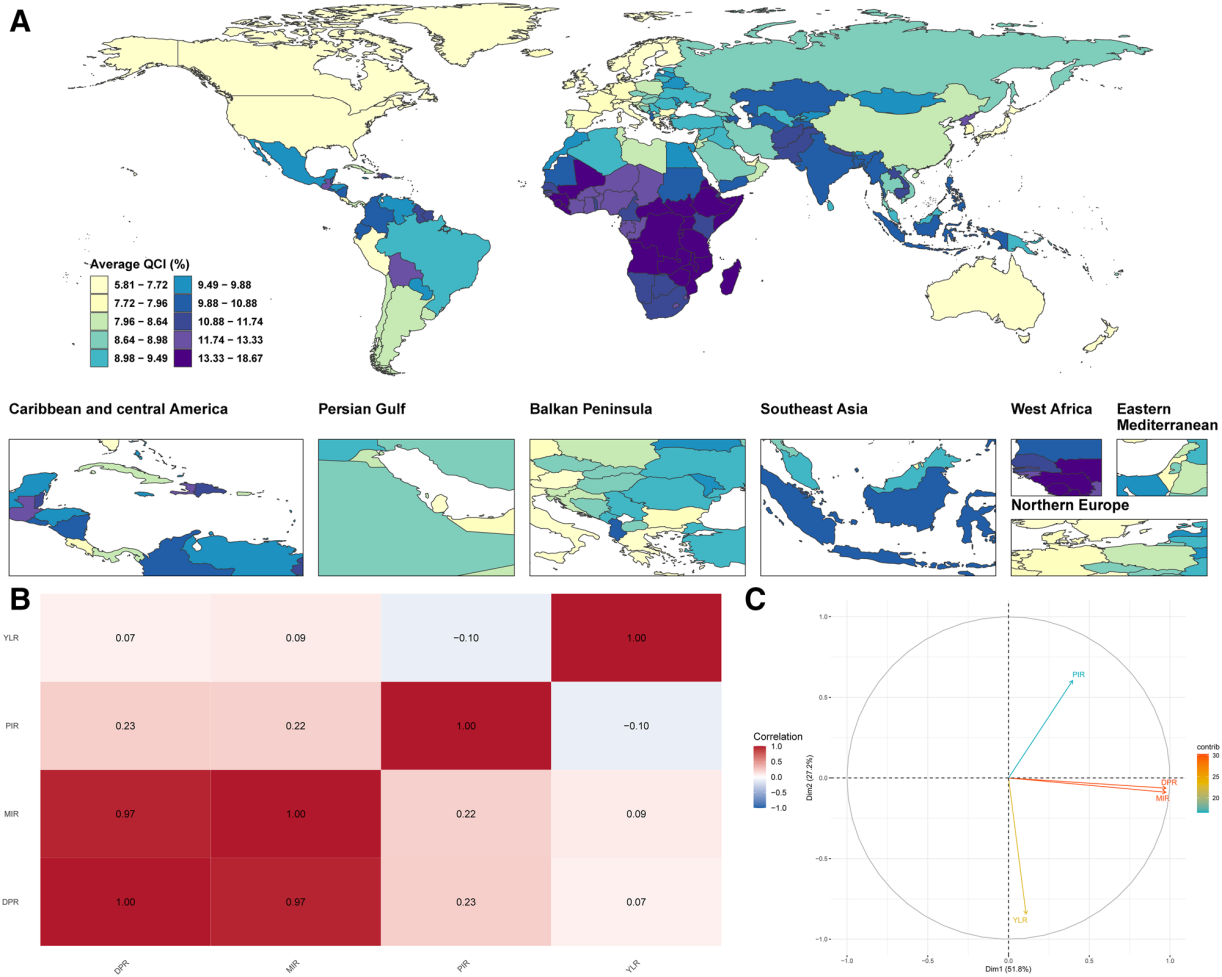
(A) Map of QCI for 204 countries. (B) Correlation of country QCI. (C) PCA of country QCI. DPR = disability-adjusted life year and prevalence ratio, MIR = mortality and incidence ratio, PCA = principal component analysis, PIR = prevalence and incidence ratio, QCI = Quality of Care Index, YLR = life lost and disability-free survival ratio.

We used the QCI equation to calculate the QCI for different countries and regions. According to the national QCI map (Fig. 1A), there are significant geographical distribution differences in the global medical quality index (QCI) for protein-energy malnutrition. The QCI in the Nordic region (Northern Europe) is the highest (13.33–18.67%), while in parts of West Africa and Southeast Asia, the QCI is the lowest (5.81–7.72%). Regions such as the Caribbean and Central America, the Persian Gulf, and the Balkan Peninsula are at a medium level (7.72–11.74%). This distribution pattern is highly consistent with the levels of socioeconomic development and the distribution of medical and health resources.

#### 5.2.2. Strong correlation with mortality indicators, weak correlation with disability measures

The correlation matrix (Fig. 1B) indicates that at the country level, QCI is strongly positively correlated with mortality-related indicators (DPR and MR, with correlation coefficients of 1.00 and 0.97, respectively). QCI shows a weak positive correlation with prevalence (PIR, 0.23) and a very weak correlation with Years Lived with Disability (YLR, 0.07). This suggests that improvements in QCI are primarily associated with reducing direct mortality from protein-energy malnutrition while having a more limited impact on disability-related burden.

#### 5.2.3. PCA validates dominance of mortality dimension

The principal component analysis (PCA) plot (Fig. 1C) shows that the first 2 principal components collectively explain 79.0% of the variance (Dim1: 51.8%, Dim2: 27.2%). Mortality indicators (MR, DPR) have very high positive loadings on Dim1, indicating that the first principal component primarily represents the mortality burden dimension. Prevalence (PIR) and years lived with disability (YLR) load higher on Dim2, representing the morbidity and disability dimension. The close clustering of QCI with mortality indicators (DPR, MR) along Dim1 provides strong confirmation that healthcare quality at the country level is predominantly reflected in mortality control.

### 5.3. The research results of QCI analysis on protein-energy malnutrition

#### 5.3.1. QCI in different SDI regions

**Figure 2.**
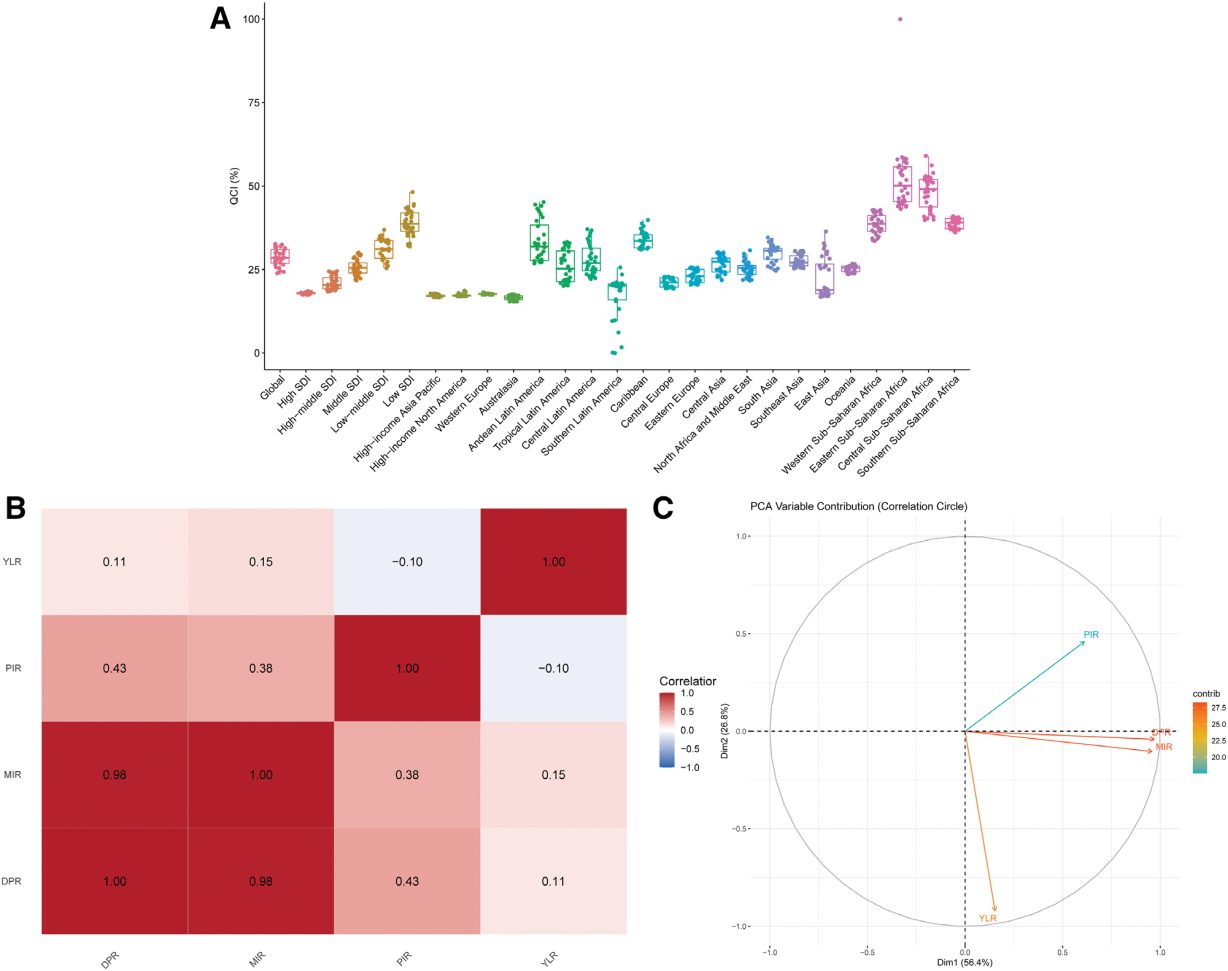
(A) Boxplot of region QCI. (B) Correlation of region QCI. (C) PCA region QCI. DPR = disability-adjusted life year and prevalence ratio, MIR = mortality and incidence ratio, PCA = principal component analysis, PIR = prevalence and incidence ratio, QCI = Quality of Care Index, SDI = Socio-Demographic Index, YLR = life lost and disability-free survival ratio.

According to the regional QCI boxplot (Fig. 2A), there are significant differences in QCI among different SDI regions. The medical quality index (QCI) for protein-energy malnutrition shows significant differences among different socioeconomic indices SDI regions. The median QCI in high SDI regions (such as high-income North America, Western Europe, and high-income Asia-Pacific) is significantly higher than that in low SDI regions (such as sub-Saharan Africa and South Asia), which is approximately 75 to 100 and 0 to 25 respectively. The global median QCI is approximately 50%, while the QCI in middle and low SDI regions is generally below 25%, indicating significant regional inequality in medical quality.

#### 5.3.2. Correlation between QCI components

Figure 2B displays the correlation matrix between the 4 components of QCI: mortality-to-incidence ratio (MIR), prevalence-to-incidence ratio (PIR), disability-adjusted life years (DALYs)-to-prevalence ratio (DPR), and years lived with disability (YLDs)-to-DALYs ratio (YLR). The analysis reveals a very strong positive correlation between MIR and DPR (*r* = 0.98), suggesting that these 2 metrics convey highly similar information about healthcare outcomes related to fatal burden and overall disease impact. PIR showed moderate positive correlations with both MIR (*r* = 0.38) and DPR (*r* = 0.43). Conversely, YLR showed very weak or negligible correlations with the other 3 components (*r* = −0.10 to 0.15), indicating it measures a distinct aspect of the healthcare burden not captured by the others, specifically related to nonfatal disability outcomes. This correlation structure suggests that QCI components can be grouped into 2 main dimensions: 1 representing fatal outcomes (MIR and DPR) and another representing disability outcomes (YLR).

#### 5.3.3. Principal component analysis (PCA) of QCI components

Figure 2C presents the results of the PCA performed on the 4 QCI components, visualized through a correlation circle. The first 2 dimensions (Dim1 and Dim2) explain 56.4% and 26.8% of the total variance, respectively, cumulatively accounting for 83.2% of the variance, indicating that these 2 dimensions adequately capture the majority of information contained in the original 4 variables.

The variables MIR and DPR are very closely grouped along the positive direction of Dim1, confirming their strong correlation identified in the correlation analysis and suggesting that Dim1 represents a common dimension of fatal outcomes and overall burden. PIR also loads positively on Dim1 but to a lesser extent, indicating it shares some common variance with the fatal outcome dimension but also contains unique information.

YLR is isolated on the positive end of Dim2, which appears to represent a distinct dimension related to nonfatal disability outcomes. This separation is consistent with the weak correlations observed between YLR and the other components. The relative positions of the variables in the correlation circle indicate that Dim1 and Dim2 are essentially orthogonal (uncorrelated) dimensions, representing independent aspects of healthcare quality for protein-energy malnutrition.

This PCA clearly reduces the 4 metrics into 2 primary, interpretable components: 1 related to mortality/fatal burden (Dim1) and 1 related to disability (Dim2), providing a more parsimonious framework for understanding patterns of care quality across regions.

### 5.4. The QCI situation in each age group and gender group

**Figure 3.**
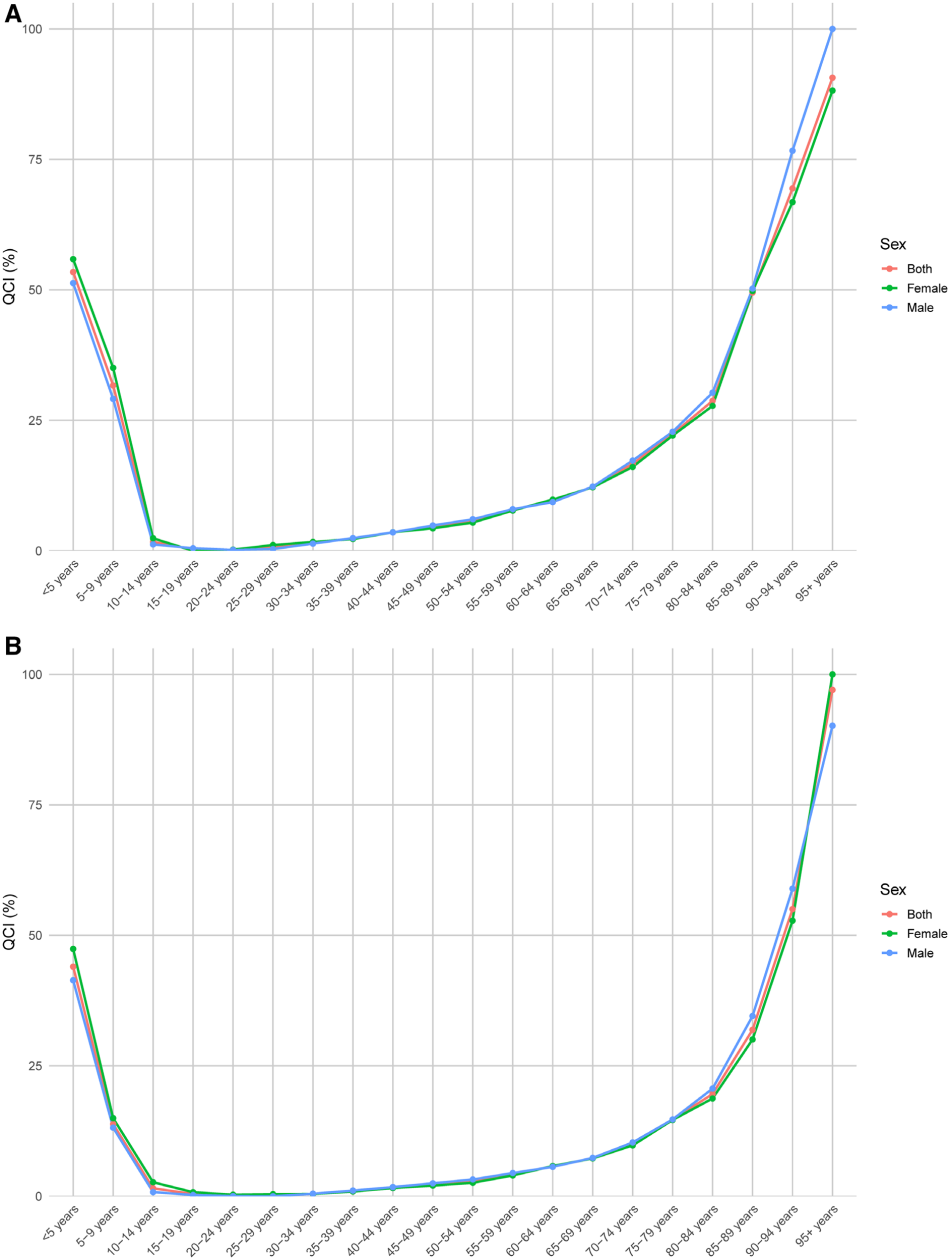
(A) The QCI situation in each age group and gender group of 1990. (B) The QCI situation in each age group and gender group of 2021. QCI = Quality of Care Index.

The Quality of Care Index (QCI) for protein-energy malnutrition demonstrated distinct age-dependent trajectories from 1990 to 2021 (Fig. 3A, B). In 1990, the highest QCI was observed in children under 5 (53.4%), which sharply declined to 31.7% (5–9 years) and reached a nadir of 1.7% (10–14 years). A gradual recovery commenced from age 15 to 19, with a sustained upward trend beginning at age 50 and accelerating notably after 65, peaking at 90.6% to 100% in individuals aged ≥85 years.

By 2021, overall QCI values declined across all age groups, with the most substantial reductions occurring in younger populations. The under-5 group decreased to 43.98% (−9.4 percentage points), while the 5 to 9 and 10 to 14 year groups fell to 13.83% and 1.52%, respectively. Although the characteristic U-shaped age pattern persisted, older age groups exhibited significant absolute reductions compared to 1990, particularly the 85 to 89 group (49.49% to 31.90%) and the 90 to 94 group (69.43% to 55.01%). The highest QCI in 2021 (97.02%) was recorded in the ≥95 years group.

Sex-specific disparities, though generally modest, demonstrated dynamic patterns across age strata. In 1990, females under 5 showed higher QCI than males (55.9% vs 51.3%), while this pattern reversed in the oldest ages, with males exhibiting superior QCI in the 90 to 94 (76.7% vs 66.8%) and ≥95 years groups (100% vs 88.2%). By 2021, the sex disparity among the youngest persisted (47.39% vs 41.38%), while a striking reversal occurred in the oldest cohort, with females reaching 100% versus 90.16% in males. A notable gender inequality emerged in the 25 to 29 year group (0.40% in females vs 0% in males).

These findings reveal persistent but evolving age and sex disparities in the quality of care for protein-energy malnutrition over 3 decades, suggesting complex interactions between demographic factors and temporal trends.

### 5.5. The SHAP analysis result

#### 5.5.1. Excellent model performance

**Figure 4.**
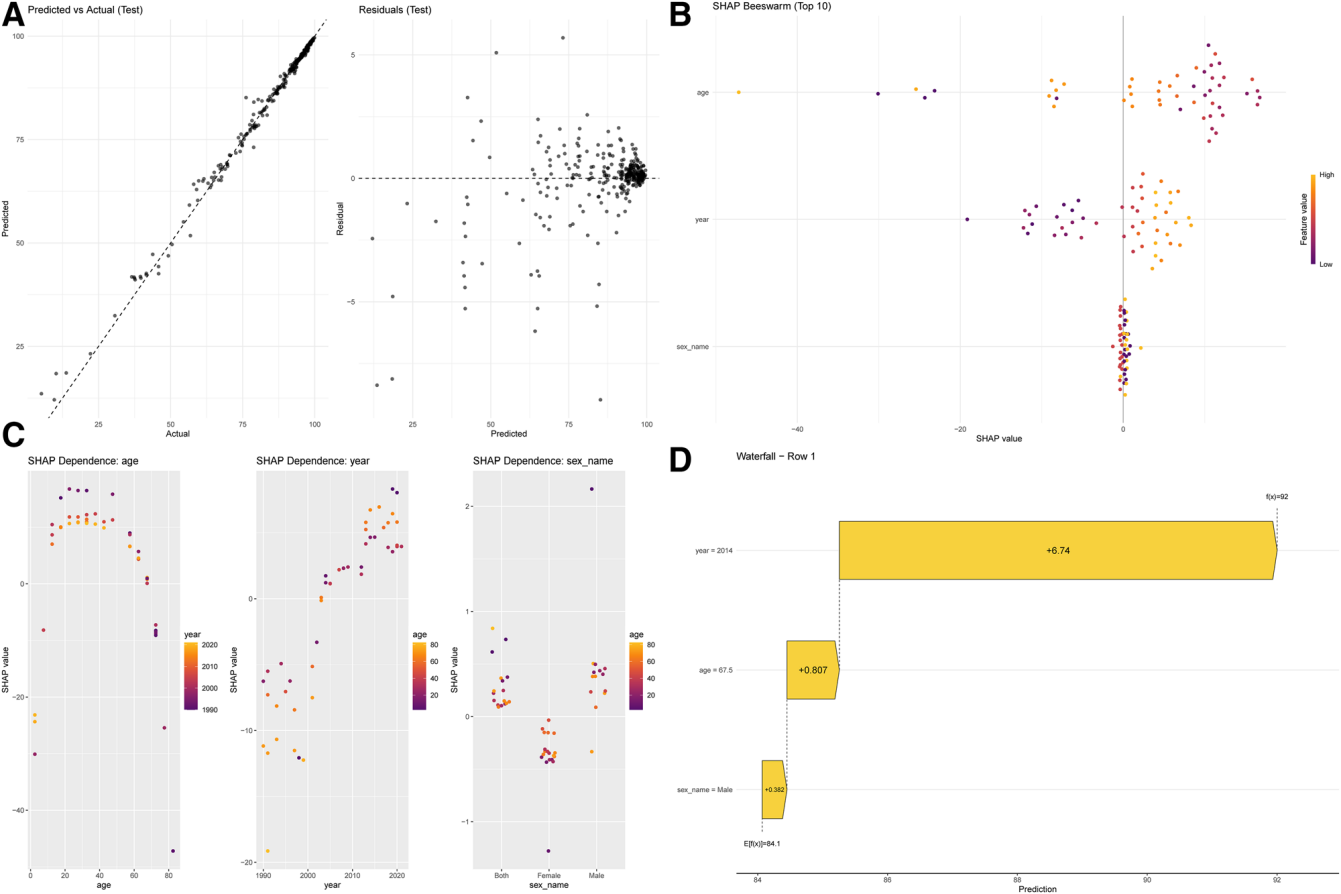
(A) Scatter plot of predicted values versus actual values. (B) SHAP Beewarm of QCI. (C) SHAP dependence (age, year, sex) of QCI. (D) The waterfall plot of QCI. QCI = Quality of Care Index, SHAP = SHapley Additive exPlanations.

The stochastic population model demonstrated exceptional performance in predicting the QCI for protein-energy malnutrition. The training set achieved an RMSE of 1.089 and R^2^ of 0.997, while the test set showed an RMSE of 1.504 and *R*^2^ of 0.994, indicating high predictive accuracy and generalization capability. The scatter plot of predicted versus actual values revealed data points closely distributed along the diagonal, with uniform residual distribution, further validating the model’s reliability (Fig. 4A).

#### 5.5.2. Feature value

SHAP analysis revealed that year was the most important feature influencing QCI, with the widest range of SHAP values (−19.14 to +8.33). The contribution of year to QCI showed a clear upward trend over time, with SHAP values generally positive and higher after 2010, indicating significant improvements in healthcare quality in recent years. Meanwhile, a significant nonlinear relationship exists between age and QCI. Middle-aged groups (35–55 years) showed the highest SHAP values (up to +16.49), indicating optimal healthcare quality for this age range. Both very young (<5 years) and very old (>75 years) populations demonstrated negative SHAP values (as low as −47.20), revealing relatively poorer healthcare quality for these age groups that require special attention. The influence of gender on QCI is relatively weak but in the same direction. The male gender usually brings a positive SHAP value (average +0.35), while the female gender usually results in a negative SHAP value (average −0.32), and the “Both” gender is in the middle position. This indicates that there may be slight gender differences in the medical quality of protein-energy malnutrition (Fig. 4B, C).

#### 5.5.3. Individual prediction interpretation reveals feature interactions

The waterfall plot showed that for a typical individual (67.5-year-old male, 2014), year contributed + 6.74 SHAP value, age contributed + 0.81, and gender contributed + 0.38. The base prediction *E*[*f*(*x*)] was 84.1, with a final prediction of 92.0, indicating this individual belonged to a high-QCI group. Analysis of SHAP values across different feature combinations revealed complex interaction effects between age, time, and gender (Fig. 4D).

### 5.6. The prediction of QCI

#### 5.6.1. Long-term trends in age-standardized QCI (1990–2033)

The age-standardized medical quality index (QCI) for global protein-energy malnutrition showed a significant upward trend from 1990 to 2021, and it is predicted to continue to increase until 2033. It rose from 17.92% (Both) in 1990 to 84.17% in 2021, an increase of approximately 66 percentage points. The prediction indicates that by 2033, the QCI will further rise to 87.65%.

#### 5.6.2. Sex disparities over time

Throughout the observation period, the age-standardized QCI for men was generally higher than that for women. In 1990, the QCI for men was 23.62%, and for women, it was 14.30% (in 1991), with a significant difference. Although both showed an upward trend, the gender gap persisted. The prediction indicates that by 2033, the QCI for women will reach 88.90%, while the data for men are not complete, but historical trends suggest that the QCI for men may still be slightly higher than that for women.

#### 5.6.3. Key historical fluctuations

There are obvious fluctuations in the data. For instance, in 1995 (Both: 15.72%), 2002 (Both: 39.58%), and 2011 (Both: 46.33%; Female: 37.77%), there were abnormally low values, which might reflect changes in data collection methods, major public health events, or the impact of model adjustments.

#### 5.6.4. Forecast trends (2022–2033)

**Figure 5.**
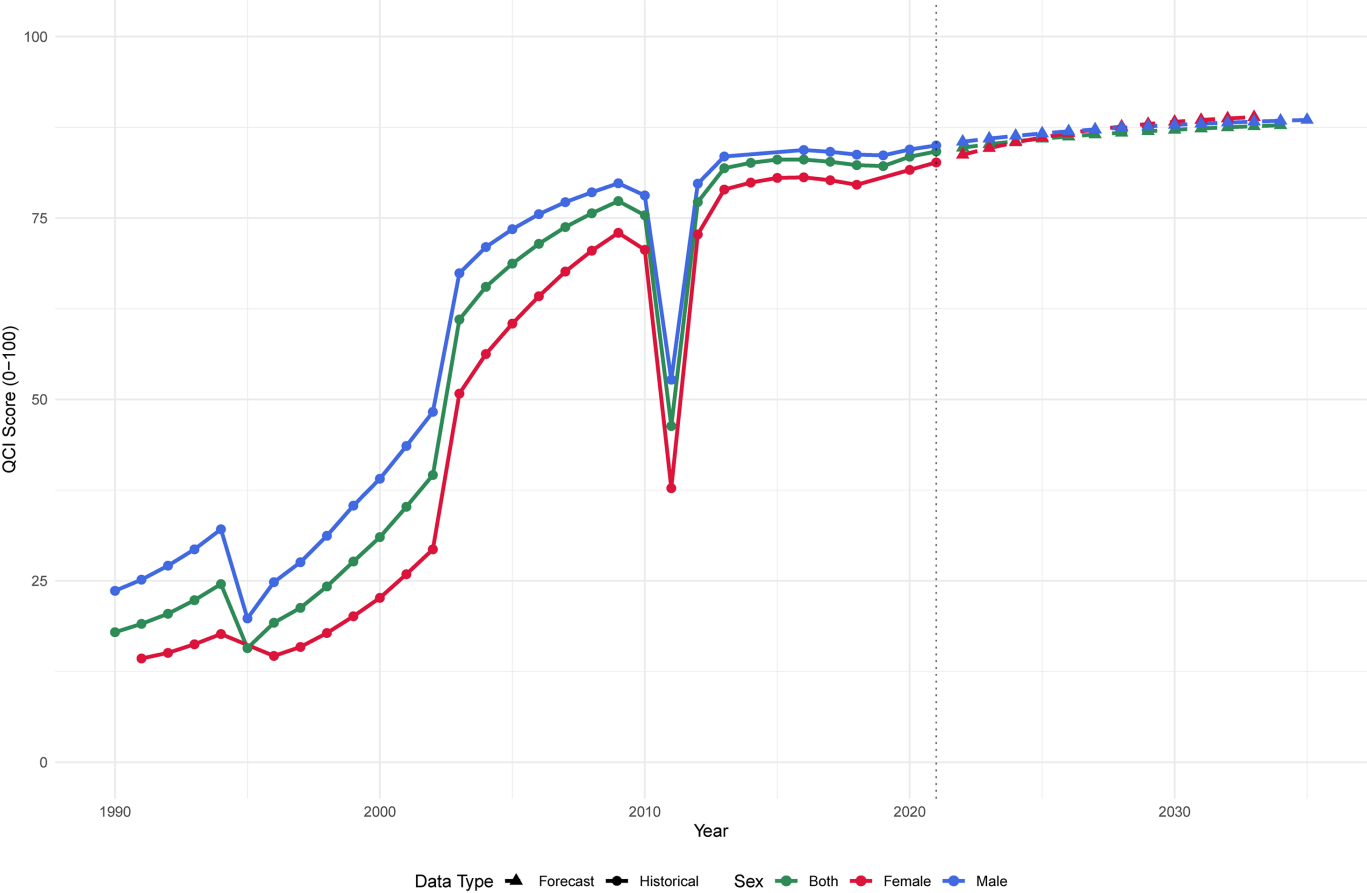
The prediction of QCI. QCI = Quality of Care Index.

The prediction based on the random population model indicates that during the period from 2022 to 2033, the global age-standardized QCI will continue to grow slowly, rising from 84.70% to 87.65%, with an average annual growth rate of approximately 0.26 percentage points. The growth of female QCI is slightly higher than the overall level, and it is expected to increase from 83.72% in 2022 to 88.90% in 2033 (Fig. 5).

### 5.7. Protein-energy malnutrition GBD prediction

#### 5.7.1. Overall trend of disease burden (1990–2035)

**Figure 6.**
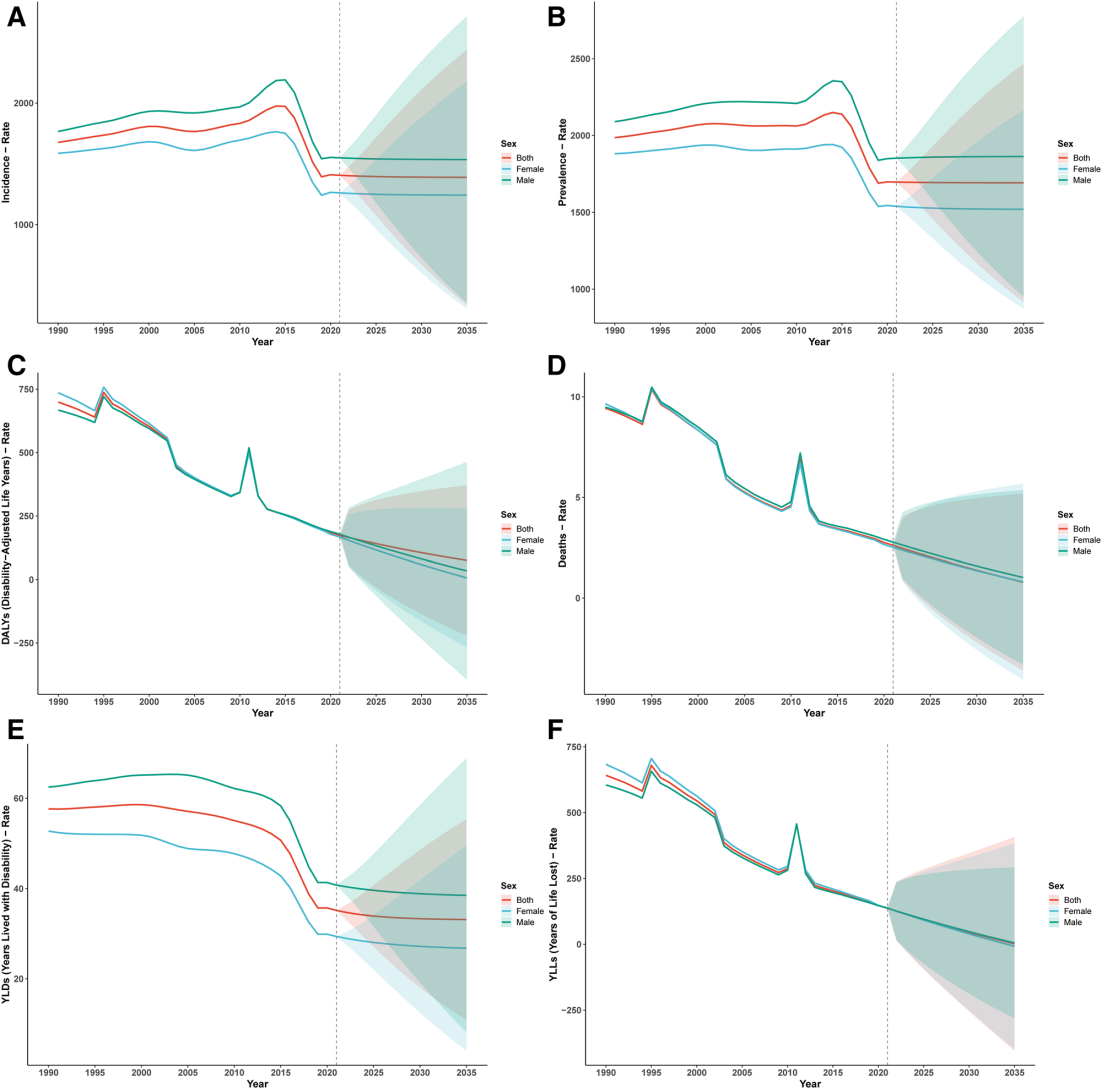
(A) Protein-energy malnutrition incidence rate prediction. (B) Protein-energy malnutrition prevalence rate prediction. (C) Protein-energy malnutrition DALYs (disability-adjusted life years) rate prediction. (D) Protein-energy malnutrition deaths rate prediction. (E) Protein-energy malnutrition YLDs (years lived with disability) rate prediction. (F) Protein-energy malnutrition YLLs (years of life lost) rate prediction. DALYs = disability-adjusted life years, YLDs = years lived with disability, YLLs = years of life lost.

Based on the analysis of GBD data and the random population model, it was shown that from 1990 to 2021, the age-standardized incidence rate, prevalence rate, and mortality rate of protein-energy malnutrition globally showed a significant downward trend (Fig. 6A, B, D). The DALYs (disability-adjusted life years) decreased from 699.30 per 1,00,000 in 1990 to 172.38 per 1,00,000 in 2021, a reduction of 75.3% (Fig. 6C). YLDs (years lived with disability) and YLLs (years of life lost) also showed similar downward trends, indicating a reduction in the overall burden of diseases (Fig. 6E, F).

#### 5.7.2. Gender differences

The age-standardized incidence rate, prevalence rate, and DALYs rate of males were higher than those of females in most years. For instance, in 2021, the DALYs rate of males was 178.38 per 1,00,000, while that of females was 166.96 per 1,00,000. The gender difference in YLLs was more obvious, indicating that males had a heavier disease burden due to premature death. However, the gender difference in YLDs was smaller, suggesting that males and females were relatively similar in terms of the disability burden (Fig. 6E, F).

#### 5.7.3. Trend prediction (2022–2035)

The stochastic life model predicts that the incidence, prevalence, and mortality rates of global protein-energy malnutrition will continue to decline slowly over the period from 2022 to 2035. The DALYs rate is expected to decrease from 165.04 per 1,00,000 in 2022 to 75.16 per 1,00,000 in 2035, with an average annual decline rate of approximately 4.5%. YLLs and YLDs also show a continuous downward trend, but the decline rate of YLDs is slightly slower than that of YLLs, suggesting that the relative importance of the disability burden may increase.

#### 5.7.4. Uncertainty analysis

The prediction intervals indicate substantial uncertainty in long-term burden projections, exemplified by the broad 95% interval of 55.68 to 274.39 per 1,00,000 for the 2035 global DALYs rate. Such wide bounds arise from 3 overlapping sources: random sampling uncertainty embedded in cross-country GBD input data, fixed age-effect constraints required for BAPC identifiability, and accumulated stochastic error when extrapolating period and cohort trends over more than a decade. The prediction interval for YLLs is especially broad, signifying greater volatility in forecasts of the premature mortality burden. Despite large uncertainty bands, the central trend estimates consistently show sustained declines in PEM-related burden metrics, which represent the reliable core signal of our projections.

## 6. Discussion

This study, based on the Global Burden of Disease (GBD) data, systematically analyzed the incidence, prevalence, and mortality of protein-energy malnutrition (PEM) worldwide and in various regions from 1990 to 2021. It used the improved QCI indicator to comprehensively quantify the global quality of care for PEM.

The results showed that over the past 3 decades, the disease burden of PEM globally exhibited a complex trend in terms of quantity, with significant differences among regions with different levels of development. Although the age-standardized rates mostly showed a downward trend (as shown in the previous results), the absolute number of cases and patients with PEM globally increased, from 1990 to 2021, by 16.2% and 12.6%, respectively. This seemingly contradictory phenomenon may mainly be attributed to the growth of the global population and the acceleration of the aging process, especially in middle and low SDI regions, where the expansion of the population base and changes in the age structure offset the decline in age-standardized rates, resulting in an increase in the total number of cases. This is consistent with the conclusions of several recent studies on the burden of malnutrition diseases, suggesting that when assessing the burden of diseases, both absolute numbers and standardized rates should be considered.^[24]^ Secondly, the distribution of the burden of PEM shows a significant socioeconomic gradient. The absolute values of the incidence, prevalence, and mortality of PEM are the highest in middle and low SDI regions, and the decline in mortality rate and DALY rate is the most significant, indicating that these regions have made significant progress in the prevention and treatment of PEM, possibly due to the nutrition improvement programs implemented globally over the past few decades, the expansion of maternal and child health care services, and poverty alleviation measures.^[25]^ However, the burden of disease in these regions remains heavy, suggesting that future public health actions still need to focus on these regions and address the fundamental social determinants of PEM, such as poverty, lack of food security, and insufficient accessibility of medical services. It is worth noting that the number of cases, patients, and deaths in high SDI regions has all shown an upward trend. This phenomenon may be related to the aging population, the dual burden of obesity and malnutrition, and changes in dietary structure in these regions. In recent years, the number of hospitalizations and deaths related to malnutrition has increased in high-income countries, especially among the elderly and patients with chronic diseases, which is referred to as the “new face of malnutrition.”^[26]^ This suggests that high SDI countries need to reevaluate their nutrition policies and strengthen screening and nutritional support for vulnerable populations. In low-resource regions, nutrition supplementation programs should be expanded, food safety should be improved, and breastfeeding should be promoted. Multiple lines of evidence confirm QCI primarily reflects care quality rather than inherent PEM epidemiological profiles. First, QCI presents a strong positive SDI gradient, consistent with cross-regional healthcare resource disparities. Second, QCI’s upward long-term trend aligns with global rollout of standardized malnutrition management guidelines and reduced PEM mortality. Third, the U-shaped age QCI pattern arises from uneven nutrition service coverage, not disease natural history. Fourth, SHAP analysis verifies that modifiable care-related factors dominate QCI variation, with negligible influence from intrinsic epidemiological features.

We acknowledge minor residual confounding from unmeasured population food insecurity, noted in study limitations. Collectively, spatial, temporal, age-stratified and machine learning results support QCI as a robust composite index for evaluating PEM care quality.

In terms of care quality, there are significant differences in the QCI of PEM across countries and regions. The QCI in Northern Europe is the highest, while in West Africa and parts of Southeast Asia, the QCI is the lowest. The QCI shows a significant gradient difference with SDI, with higher QCI scores in high SDI regions and lower QCI scores in low SDI regions. In regions with a high SDI (such as high-income North America, Western Europe, and high-income Asia-Pacific), the care quality index is higher. These results reflect the significant progress made by developed countries in the treatment and care of PEM. In contrast, the care quality index is significantly lower in many regions with a low SDI (such as sub-Saharan Africa and South Asia), which is consistent with the trends of disease incidence, prevalence, and mortality. This highlights that the level of social and economic development is an important factor determining the quality of treatment and care,^[27,28]^ emphasizing the importance and urgency of improving regional economic levels, strengthening health infrastructure construction, and increasing advanced treatment methods.^[29,30]^ This study, through national-level analysis, revealed significant geographical differences in the global QCI for protein-energy malnutrition and confirmed the high correlation between QCI and mortality control. Nordic countries demonstrated the highest medical quality, while West Africa and Southeast Asia bore the heaviest burden and had the lowest QCI. Correlation analysis and principal component analysis consistently indicated that the improvement in QCI was mainly reflected in the reduction of mortality rates, with a relatively smaller impact on disability-related burdens. Future intervention measures should target high-burden and low-QCI regions, focusing on strengthening the capacity for the treatment of acute malnutrition while developing comprehensive strategies that also take into account disability prevention.

It should be noted that the QCI established in this study only relies on disease outcome indicators without incorporating direct structural and process care metrics. However, the strong correlation between QCI and SDI indirectly reflects the role of healthcare resources and service accessibility in improving PEM care outcomes. Regions with superior medical infrastructure, adequate human resources, and universal nutrition service coverage (high SDI regions) achieve persistently higher QCI, which echoes the core logic that sound care system inputs translate into reduced mortality and disease burden. The synchronous growth of global QCI alongside the worldwide promotion of standardized PEM management guidelines also implies that optimized clinical care processes drive better population-level outcomes captured by QCI.

Furthermore, the QCI analysis revealed significant differences in the quality of protein-energy malnutrition care across different age groups. From 1990 to 2021, the QCI for children under 5 years old was at a relatively high level and continued to decline, reaching its lowest point at 10 to 14 years old, then gradually rising and reaching the highest level among the elderly at 85 years and above, even surpassing the levels during childhood. These findings highlight the significant age-related inequalities in the treatment and care of protein-energy malnutrition. This may be attributed to the increased number of studies on PEM in children and the elderly, the introduction of a series of standardized care measures, as well as the strengthening of infant and child care^[31,32]^ and the more frequent contact of the elderly with the healthcare system, chronic disease co-management, and nutrition support services.^[33-35]^ These factors have contributed to the improvement of QCI for both children and the elderly. However, the QCI levels for adolescents and adults were lower compared to those of children and the elderly, suggesting the inadequacy of PEM care for adolescents and adults during puberty. Targeted intervention measures are crucial for addressing the challenges faced by adolescents and adults with protein-energy malnutrition and promoting fair medical care for patients of all ages. Among the 10 to 14 and 15 to 19 age groups, the QCI was abnormally low, suggesting a possible severe gap in medical service quality or data reporting issues in this age range. Overall, the QCI for global protein-energy malnutrition declined across all age groups from 1990 to 2021, especially among children, adolescents, and the elderly. There was a relatively small gender difference, but it still existed, and the differences varied by age group. In the <5-year-old group, the QCI for females (55.9%) was higher than that for males (51.3%). The extremely low QCI value for adolescent males should be given high attention, indicating a serious challenge in the nutrition service for this group. In the 90 to 94 age group, the QCI for males (76.7%) was significantly higher than that for females (66.8%). In the 95+ age group, the QCI for males reached 100% and for females was 88.2%, suggesting possible gender-based medical quality inequality in the very elderly group. The significant differences between men and women in high age groups may be related to their health conditions and life expectancy, and further research is needed.

This study, based on GBD data and the analysis of the random population model, shows that the age-standardized incidence rate, prevalence rate, and mortality rate of protein-energy malnutrition have shown a significant downward trend from 1990 to 2021. This improvement is mainly attributed to the progress in treatment protocols and the significant enhancement of supportive care. The age-standardized incidence rate, prevalence rate, and DALY rate of males were higher than those of females in most years. The disease burden has significantly decreased over the past 3 decades, but the gender disparity persists, with males bearing a higher burden. The model prediction for 2022 to 2035 shows that the incidence rate, prevalence rate, and mortality rate of global protein-energy malnutrition will continue to decline slowly. This suggests that the burden will continue to decrease in the future, but there is considerable uncertainty, especially regarding premature death burden. Attention should be paid to high-risk groups and the optimization of resource allocation. Meanwhile, this study analyzed the QCI of protein-energy malnutrition from 1990 to 2021. Through the random population model, the age-standardized QCI data of global protein-energy malnutrition from 1990 to 2033 were analyzed, and the QCI trend in the next 15 years was predicted using ES analysis. The QCI has significantly improved over the past 3 decades, but there have been fluctuations. The gender disparity persists, with males’ QCI historically being higher than females’. The prediction shows that the QCI will continue to grow slowly in the future, indicating that the overall nutritional quality of malnutrition treatment globally is improving, but issues related to data fluctuations and gender inequality still need to be addressed.

The results of this study emphasize that in order to improve the quality of care for patients with protein-energy malnutrition (PEM) globally, targeted intervention measures and policy changes need to be implemented urgently, especially in regions with scarce resources and low service accessibility. Future work should focus on the most vulnerable groups. By optimizing resource allocation and establishing a fair care service system, efforts should be made to narrow the gap in care quality between different regions, ultimately ensuring that all patients with PEM can receive high-quality and affordable care services.

This study still has certain limitations. The QCI is exclusively built on epidemiological outcome data and lacks direct structural and process care indicators such as medical infrastructure, nutrition service access, clinical workforce allocation, and guideline compliance rates, which restricts its capacity to fully reflect multidimensional care quality. Uniform cross-national long-term panel data of these care process/structural metrics are not available in the GBD dataset covering 1990 to 2021. Future research will integrate standardized structural, process, and outcome indicators to develop a more comprehensive PEM care quality index when consistent global care input data become accessible.

## 7. Conclusion

Leveraging data from the Global Burden of Disease (GBD) study 1990–2021, this study presents a comprehensive analysis of the global burden and quality of care for protein-energy malnutrition (PEM), utilizing the Quality of Care Index (QCI). Our findings indicate that despite a continued decline in age-standardized incidence, prevalence, and mortality rates, the absolute number of PEM cases has risen, driven by population growth and aging. The disease burden remains disproportionately high in low and middle Socio-Demographic Index (SDI) regions, where health systems are often under-resourced. Furthermore, significant socioeconomic and geographic disparities persist in both PEM burden and quality of care. Simultaneously, high-SDI regions are confronting a complex dual burden of undernutrition and obesity, underscoring the evolving challenge of PEM across development settings.

## Acknowledgments

We would also like to thank the countless individuals who have contributed to the Global Burden of Disease Study 2021 in various capacities.

## Author contributions

**Conceptualization:** Yaxue Chen, Hongjiang Pu.

**Data curation:** Yaxue Chen, Hongjiang Pu.

**Formal analysis:** Yaxue Chen.

**Investigation:** Wei Chen.

**Methodology:** Wei Chen.

**Project administration:** Jia Liu, Min Deng.

**Resources:** Yaxue Chen, Jia Liu, Min Deng, Li Hu.

**Software:** Li Hu, Ying Ye.

**Supervision:** Li Hu, Ying Ye.

**Validation:** Yaxue Chen, Jingwen Zhang, Ying Ye, Hongjiang Pu.

**Visualization:** Yaxue Chen, Jingwen Zhang, Hongjiang Pu.

**Writing – original draft:** Yaxue Chen, Jingwen Zhang, Hongjiang Pu.

**Writing – review & editing:** Yaxue Chen, Ying Ye, Hongjiang Pu.
